# Supplementary material for: Content-rich biological network constructed by mining PubMed abstracts
Source: BMC Bioinformatics. 2004 Oct 8;5:147. doi: 10.1186/1471-2105-5-147 (PMC528731; doi:10.1186/1471-2105-5-147)
Supplement: Additional File 2 — The original results of the above study (non-essential files are deleted to keep the file size under the limit set by BMC bioinformatics). [file 1471-2105-5-147-S2.bz2 › chilibotAdditionalFile2/dip40/324ID8754798E3494/html/NFYB_NFYA.html]

 


 **NFYB** and **NFYA** 
  
Found 81 abstracts in PubMed, retrieved 40.  
 

 What does Google say? 
 PDF only 
| .edu only 

---

**Interactive relationship** (e.g. stimulation, inhibition, etc)

**Neutral relationship**- ATF6alpha and ATF6beta produced by ER stress induced proteolysis bind to CCACG only when CCAAT is bound to NF Y, a heterotrimer consisting of NF YA  [ **NFYA** ] , NF YB  [ **NFYB** ] , and NF YC.  Ref: 11158310 Mol Cell Biol, 2001
- The results unexpectedly demonstrated that in rat mammary cells CArG box binding factor A CBF A  [ **NFYA** ]   [ **NFYB** ]  is the major protein species that bind specifically to the rat and human HRE sequences with high affinity.  Ref: 11000268 Nucleic Acids Res, 2000
- and iii the interactions between NF YA  [ **NFYA** ] , NF YB  [ **NFYB** ]  NF YC, and H3 H4 or nucleosomes are not mutually exclusive.  Ref: 10567583 Mol Cell Biol, 1999
- In contrast, expression of an allele specific CBF B  [ **NFYA** ]   [ **NFYB** ]  mutant that binds high affinity to a mutant CBF binding site containing a CCAAC motif revives transcription activation of an inactive mutant topo II alpha promoter containing CCAAC during the cell cycle.  Ref: 12149265 J Biol Chem, 2002
- By expressing epitope tagged isoforms of CBF A  [ **NFYA** ]   [ **NFYB** ]  it was shown that the p42 isoform binds the COX 2 ARE in EMSA of crude cell extracts.  Ref: 12086581 Biochem J, 2002
- Altogether, these results indicated that the specific DNA contact surface of each CBF subunit is generated as a result of interaction between CBF B  [ **NFYA** ]   [ **NFYB** ]  and CBF A  [ **NFYA** ]   [ **NFYB** ]  CBF C heterodimer.  Ref: 9813075 J Biol Chem, 1998
- This region specifically bound to nuclear proteins extracted from rat hearts, and gel shift assays using competitive oligonucleotides, antibodies and recombinant proteins showed that this region binds to the NF YA  [ **NFYA** ]  and NF YB  [ **NFYB** ] , members of the CCAAT binding nuclear protein family.  Ref: 9874243 Eur J Biochem, 1998
- CBF A  [ **NFYA** ]   [ **NFYB** ]  and mElfin are known to bind to cytoskeletal F actin.  Ref: 12639895 Endocrinology, 2003
- A deletion analysis suggests that a segment of CBF B  [ **NFYA** ]   [ **NFYB** ]  needed for transcription activation is also involved in interaction with dTAF110.  Ref: 9512492 Biochem J, 1998
- CBF B  [ **NFYA** ]   [ **NFYB** ]  and CBF C were cross linked more strongly than CBF A  [ **NFYA** ]   [ **NFYB** ] .  Ref: 9813075 J Biol Chem, 1998
- A subunit interaction domain of NF YA  [ **NFYA** ] , which forms a heterotrimer with NF YB  [ **NFYB** ]  and NF YC, is not required for these interactions with Sp1 or Sp3.  Ref: 10748033 J Biol Chem, 2000
- The X element binds RFX RFX5 RFXANK RFXB RFXAP and CREB, while Y binds NF Y CBF NF YA  [ **NFYA** ] , NF YB  [ **NFYB** ] , and NF YC.  Ref: 10913187 Mol Cell Biol, 2000
- The affinity of CBF A  [ **NFYA** ]   [ **NFYB** ]  binding to HRE was significantly higher than to the CArG box described as a recognition sequence for CBF A  [ **NFYA** ]   [ **NFYB** ]  protein.  Ref: 11000268 Nucleic Acids Res, 2000
- The histone fold motifs of CBF A  [ **NFYA** ]   [ **NFYB** ]  and CBF C interact with each other to form a heterodimer that associates with CBF B  [ **NFYA** ]   [ **NFYB** ]  to form a heterotrimeric CBF molecule, which then binds to DNA.  Ref: 9612081 Trends Biochem Sci, 1998
- No subunit of heterotrimeric CBF containing DNA binding domain mutant of either CBF B  [ **NFYA** ]   [ **NFYB** ]  or CBF C was cross linked to DNA, and interestingly, cross linking of CBF B  [ **NFYA** ]   [ **NFYB** ]  that occurred without CBF A  [ **NFYA** ]   [ **NFYB** ]  CBF C was inhibited in presence of mutant CBF C CBF A  [ **NFYA** ]   [ **NFYB** ]  heterodimer.  Ref: 9813075 J Biol Chem, 1998
- This element bound specific complexes containing NF YA  [ **NFYA** ]  and NF YB  [ **NFYB** ]  proteins in SH SY5Y nuclear extracts, the binding of which was augmented by 5 AzaC in association with enhanced levels of NF YB  [ **NFYB** ]  protein and the function of which was confirmed by inhibition using dominant negative NF YA  [ **NFYA** ] .  Ref: 12749850 Exp Cell Res, 2003

**Non-interactive relationship** (e.g. studied together, co-existance, homology, etc.)

- Using a mammalian two hybrid system in HeLa cells, it was shown that both Sp1 and Sp3 interacted with NF YA  [ **NFYA** ]  but not NF YB  [ **NFYB** ]  and NF YC.  Ref: 10748033 J Biol Chem, 2000
- In contrast, in an electrophoretic mobility shift assay of crude RAW 264.7 macrophage like cell extracts, an antiserum that recognizes both AUF1 and CBF A  [ **NFYA** ]   [ **NFYB** ]  failed to supershift complexes formed on the TNF alpha ARE, but did supershift a complex specific for the COX 2 ARE.  Ref: 12086581 Biochem J, 2002
- In addition, the transient relationship between CBV T and CBF A  [ **NFYA** ]   [ **NFYB** ]  is region specific.  Ref: 12465108 Magn Reson Med, 2002
- The transient relationship between arterial cerebral blood flow CBF A  [ **NFYA** ]   [ **NFYB** ]  and total cerebral blood volume CBV T was determined in the rat brain.  Ref: 12465108 Magn Reson Med, 2002
- Taken together, our results suggest that CBF A  [ **NFYA** ]   [ **NFYB** ]  may play an important role in transcriptional regulation of Ha ras promoter activity during normal mammary cell growth and carcinogenesis.  Ref: 11000268 Nucleic Acids Res, 2000
- In this study, by using a series of deletion mutations of CBF B  [ **NFYA** ]   [ **NFYB** ]  and CBF C in transcription assay in vitro, we further delineated smaller segments in these domains that were sufficient to support transcriptional activation by CBF.  Ref: 9512492 Biochem J, 1998
- The relationship between CBV T and CBF A  [ **NFYA** ]   [ **NFYB** ]  under transient conditions is similar to the power law under steady state conditions.  Ref: 12465108 Magn Reson Med, 2002
- The role of CBF in activating transcription was further substantiated by inhibition of promoter activity with a plasmid expressing a dominant negative CBF B  [ **NFYA** ]   [ **NFYB** ]  mutant.  Ref: 11704838 Oncogene, 2001
- NF Y is composed of three subunits, NF YA  [ **NFYA** ] , NF YB  [ **NFYB** ] , and NF YC, all required for DNA binding.  Ref: 12857858 Mol Biol Cell, 2003
- The amino acid sequence of CBF A  [ **NFYA** ]   [ **NFYB** ]  is highly similar to that of the ARE binding protein AUF1.  Ref: 12086581 Biochem J, 2002
- This showed that at equilibrium DNA binding conditions, the CBF A  [ **NFYA** ]   [ **NFYB** ]  CBF C heterodimer is very stable, but association between CBF B  [ **NFYA** ]   [ **NFYB** ]  and CBF A  [ **NFYA** ]   [ **NFYB** ]  CBF C is very weak.  Ref: 9813075 J Biol Chem, 1998
- In each CBF subunit, the segment needed for formation of the CBF DNA complex is conserved from yeast to human and, interestingly, the conserved segment of two CBF subunits, CBF A  [ **NFYA** ]   [ **NFYB** ]  and CBF C, are homologous to the histone fold motif of eukaryotic histones and archaebacterial histone like protein HMf 2.  Ref: 9612081 Trends Biochem Sci, 1998
- We conclude that specificity of the NF Y trimer is not due to NF YA  [ **NFYA** ]  only, but stems in part from the contribution of the histone fold alpha1, particularly that of NF YB  [ **NFYB** ] .  Ref: 9973554 J Mol Biol, 1999
- Recombinant CBF A  [ **NFYA** ]   [ **NFYB** ]  bound the COX 2 and TNF alpha AREs, but not a non specific control RNA.  Ref: 12086581 Biochem J, 2002
- In addition, unidentified proteins also bound the Oct motif specifically in the absence of CBF A  [ **NFYA** ]   [ **NFYB** ]  binding.  Ref: 12427543 Biochim Biophys Acta, 2002
- We report that relative to each agent individually, successive incubation of G 2 d CGG n with TMPyP4 followed by exposure to CBF A  [ **NFYA** ]   [ **NFYB** ]  results in a nearly additive extent of disruption of this tetraplex form of the repeat sequence.  Ref: 12853612 Nucleic Acids Res< MedlineTA>Nucleic Acids Res, 2002
- in contrast, CBF B  [ **NFYA** ]   [ **NFYB** ]  was cross linked in the absence of CBF A  [ **NFYA** ]   [ **NFYB** ]  CBF C.  Ref: 9813075 J Biol Chem, 1998
- It is a trimer formed by NF YA  [ **NFYA** ]  and two putative histone like subunits, NF YB  [ **NFYB** ]  and NF YC, showing similarity to histones H2B and H2A, respectively.  Ref: 9880503 J Biol Chem, 1999
- We clone Xenopus NF YA  [ **NFYA** ] , NF YB  [ **NFYB** ]  and NF YC and establish that NF Y is the predominant Y box binding protein in Xenopus oocyte nuclei.  Ref: 9799238 EMBO J, 1998
- The nature of the association of CBF B  [ **NFYA** ]   [ **NFYB** ]  with CBF A  [ **NFYA** ]   [ **NFYB** ]  CBF C was also revealed by studying the inhibition of CBF DNA complex formation by the mutant CBF B  [ **NFYA** ]   [ **NFYB** ] .  Ref: 9813075 J Biol Chem, 1998
- Gel shift assays using either single or double mutations of the Oct and CCAAT sequences as well as super shift assays demonstrated single and double occupancy of these two sites by Oct 1 and CBF A  [ **NFYA** ]   [ **NFYB** ] .  Ref: 12427543 Biochim Biophys Acta, 2002
- CBF a  [ **NFYA** ]   [ **NFYB** ]  biophysical perspective.  Ref: 11105902 Semin Cell Dev Biol, 2000
- For the measurement of cerebral blood flow CBF a  [ **NFYA** ]   [ **NFYB** ]  modified Kety Schmidt saturation technique with argon as inert tracer gas was used.  Ref: 9813515 Br J Anaesth, 1998
- Differentially expressed proteins were analyzed by matrix assisted laser desorption ionization time of flight mass spectrometry and two androgen regulated proteins were identified as mElfin and CArG binding factor A CBF A  [ **NFYA** ]   [ **NFYB** ] .  Ref: 12639895 Endocrinology, 2003
- Previous studies have shown that CBF contains two transcription activation domains a glutamine rich, serine threonine rich domain present in the CBF B  [ **NFYA** ]   [ **NFYB** ]  subunit and a glutamine rich domain in the CBF C subunit.  Ref: 9512492 Biochem J, 1998
- In fact, CBF A  [ **NFYA** ]   [ **NFYB** ]  expressed from its cDNA was indistinguishable from protein D2 in molecular size and immunoreactivity to McAb 351.  Ref: 11432731 Eur J Biochem, 2001
- Based on peptide sequence homology of qTBP42 and mouse CArG box binding factor A CBF A  [ **NFYA** ]   [ **NFYB** ] , we provide direct evidence that recombinant CBF A  [ **NFYA** ]   [ **NFYB** ]  protein is physically and immunochemically indistinguishable from qTBP42.  Ref: 12202751 Nucleic Acids Res, 2002
- To understand the in vivo function of CBF, a dominant negative mutant of CBF B  [ **NFYA** ]   [ **NFYB** ]  subunit that inhibits DNA binding of wild type CBF was stably expressed in mouse fibroblast cells under control of tetracycline responsive promoter.  Ref: 10660616 J Biol Chem, 2000
- CArG binding factor A CBF A  [ **NFYA** ]   [ **NFYB** ]  is involved in transcriptional regulation of the rat Ha ras promoter.  Ref: 11000268 Nucleic Acids Res, 2000
- Our observations open up the prospect of unfolding secondary structures of the expanded FMR1 d CGG n tract of fragile X cells by their exposure to low molecular size drugs or to proteins such as TMPyP4 or CBF A  [ **NFYA** ]   [ **NFYB** ] .  Ref: 12853612 Nucleic Acids Res< MedlineTA>Nucleic Acids Res, 2000
- These two proteins share an identical amino acid sequence except for a 47 residue insertion in AlF C1, and are members of a gene family including heterogeneous nuclear ribonucleoprotein hnRNP and CCAAT binding factor subunit A CBF A  [ **NFYA** ]   [ **NFYB** ]  genes.  Ref: 11245986 Gene, 2001
- NF Y is a trimeric CCAAT binding factor with histone fold subunits NF YB  [ **NFYB** ]  NF YC and bipartite activation domains located on NF YA  [ **NFYA** ]  and NF YC.  Ref: 10373572 Nucleic Acids Res, 1999
- Furthermore, overexpression of a dominant negative CBF B  [ **NFYA** ]   [ **NFYB** ]  NF YA  [ **NFYA** ]  subunit significantly inhibited promoter activity in both transient and stable cells.  Ref: 12805369 J Biol Chem< MedlineTA>J Biol Chem, 1999
- CBF A  [ **NFYA** ]   [ **NFYB** ]  exists as two isoforms, p37 and p42, that differ by a 47 amino acid insertion close to the C terminus.  Ref: 12086581 Biochem J, 2002
- We inquired whether CBF A  [ **NFYA** ]   [ **NFYB** ]  employs the same or different domains to differentially interact with G 2 d CGG n and G 2 d TTAGGG n.  Ref: 12202751 Nucleic Acids Res, 2002
- By microsequencing, protein D2 was identified with a known protein, CArG box motif binding factor A CBF A  [ **NFYA** ]   [ **NFYB** ] , which has been characterized as a transcriptional repressor, and C2 as its isoform protein.  Ref: 11432731 Eur J Biochem, 2001
- Mutant CBF A  [ **NFYA** ]   [ **NFYB** ]  proteins that lack each or combinations of its five conserved motifs RNP1 1, RNP1 2, RNP2 1, RNP2 2 and ATP GTP binding box were tested for their G 2 d CGG n destabilization and G 2 d TTAGGG n stabilization activities.  Ref: 12202751 Nucleic Acids Res, 2002
- Transient transfection assays using reporter plasmids verified that mutations within the HRE that disrupt binding of CBF A  [ **NFYA** ]   [ **NFYB** ]  also reduced the activity of the rat Ha ras promoter.  Ref: 11000268 Nucleic Acids Res, 2000
- Similarly to TMPyP4, the hnRNP related protein CBF A  [ **NFYA** ]   [ **NFYB** ]  also destabilizes G 2 tetrahelical d CGG n while binding and stabilizing tetraplex telomeric DNA.  Ref: 12853612 Nucleic Acids Res< MedlineTA>Nucleic Acids Res, 2000
- Consistent with this observation, expression of a truncated CBF B  [ **NFYA** ]   [ **NFYB** ]  subunit lacking a transcription activation domain, which inhibits activity of a collagen promoter, does not affect activity of the topo II alpha promoter in fibroblast cells.  Ref: 12149265 J Biol Chem, 2002
- Epitope tagged p42 CBF A  [ **NFYA** ]   [ **NFYB** ]  expressed in HeLa cells co immunoprecipitated with endogenous COX 2 mRNA, but not glyceraldehyde 3 phosphate dehydrogenase mRNA, as shown by reverse transcription PCR.  Ref: 12086581 Biochem J, 2002
- Posttranslational modification of mElfin and CBF A  [ **NFYA** ]   [ **NFYB** ]  by androgens may be associated with a cytoskeletal change that is involved in androgen regulated gene expression.  Ref: 12639895 Endocrinology, 2003
- The similarity between CBF A  [ **NFYA** ]   [ **NFYB** ]  and AUF1 suggests that CBF A  [ **NFYA** ]   [ **NFYB** ]  could be re named AUF2.  Ref: 12086581 Biochem J, 2002
- None of the CBF A  [ **NFYA** ]   [ **NFYB** ]  and CBF C subunits, which together formed a CBF A  [ **NFYA** ]   [ **NFYB** ]  CBF C heterodimer, were cross linked without CBF B  [ **NFYA** ]   [ **NFYB** ] .  Ref: 9813075 J Biol Chem, 1998
- ... NF Y derived from class II plasma B cells, and from bare lymphocyte syndrome group II cell lines, RJ2.2.5 and RM3, dissociated into discrete NF YA  [ **NFYA** ]  and NF YB  [ **NFYB** ]  C subunit fractions.  Ref: 9660784 J Biol Chem, 1998
- This study indicated that the association between CBF B  [ **NFYA** ]   [ **NFYB** ]  and CBF A  [ **NFYA** ]   [ **NFYB** ]  CBF C is stabilized upon interaction with DNA, a process likely to favor formation of a high affinity CBF DNA complex.  Ref: 9813075 J Biol Chem, 1998
- We now report the isolation of the genes hapB and hapE, which encode proteins with central regions of high similarity to Hap2p and Hap5p of S. cerevisiae and to the CBF B  [ **NFYA** ]   [ **NFYB** ]  and CBF C proteins of mammals.  Ref: 9858535 Mol Cell Biol, 1999
- This region dependent transcription in cardiac myocytes transfected with antisense cDNAs encoding NF YA  [ **NFYA** ]  and NF YB  [ **NFYB** ]  was decreased to approximately 50% of that seen in cells transfected with the same sense cDNAs.  Ref: 9874243 Eur J Biochem, 1998
- Hence, CBF A  [ **NFYA** ]   [ **NFYB** ]  employs different domains to destabilize G 2 d CGG n or stabilize G 2 d TTAGGG n.  Ref: 12202751 Nucleic Acids Res, 2002
- The analysis of cell growth using bromodeoxyuridine labeling showed that expression of the mutant CBF B  [ **NFYA** ]   [ **NFYB** ]  decreased the number of cells entering into S phase, and also delayed induction of S phase in the quiescent cells after serum stimulation, thus indicating that the inhibition of CBF binding prolonged the progression of S phase in fibroblasts.  Ref: 10660616 J Biol Chem, 2000
- In a HeLa cell tetracycline regulated reporter system, overexpression of the p42 CBF A  [ **NFYA** ]   [ **NFYB** ]  isoform resulted in stabilization of a COX 2 ARE reporter mRNA.  Ref: 12086581 Biochem J, 2002
- Exogenous cdk4 overcomes reduced cdk4 RNA and inhibition of G1 progression in hematopoietic cells expressing a dominant negative CBF a  [ **NFYA** ]   [ **NFYB** ]  model for overcoming inhibition of proliferation by CBF oncoproteins.  Ref: 10851069 Oncogene, 2000
- The six alternatively spliced NF YA  [ **NFYA** ]  isoforms all showed significantly less synergistic transactivation of the CBS 1b promoter with Sp1 than wild type NF YA  [ **NFYA** ] , as determined by cotransfections in Drosophila SL2 cells with NF YB  [ **NFYB** ]  and NF YC.  Ref: 12427542 Biochim Biophys Acta, 2002
- ii NF YB  [ **NFYB** ]  NF YC and NC2alpha beta do not form heterodimers and NC2 cannot associate NF YA  [ **NFYA** ] .  Ref: 9973554 J Mol Biol, 1999
- Expression of the mutant CBF B  [ **NFYA** ]   [ **NFYB** ]  but not the wild type CBF B  [ **NFYA** ]   [ **NFYB** ]  resulted in retardation of fibroblast cell growth.  Ref: 10660616 J Biol Chem, 2000
